# Correction: ACC-deaminase producing plant growth promoting rhizobacteria and biochar mitigate adverse effects of drought stress on maize growth

**DOI:** 10.1371/journal.pone.0250286

**Published:** 2021-04-12

**Authors:** The PLOS ONE Staff

There is an error in affiliation 2 for author Fauzia Mohsin. The publisher apologizes for the error.

The correct affiliation 2 is: Department of Biology, Government College for Women Chungi No. 14, Multan, Pakistan.

## References

[1] Danish S, Zafar-ul-Hye M, Mohsin F, Hussain M (2020) ACC-deaminase producing plant growth promoting rhizobacteria and biochar mitigate adverse effects of drought stress on maize growth. PLoS ONE 15(4): e0230615. 10.1371/journal.pone.0230615 32251430 PMC7135286

